# Genomic Resources of Three *Pulsatilla* Species Reveal Evolutionary Hotspots, Species-Specific Sites and Variable Plastid Structure in the Family Ranunculaceae

**DOI:** 10.3390/ijms160922258

**Published:** 2015-09-15

**Authors:** Monika Szczecińska, Jakub Sawicki

**Affiliations:** 1 Department of Botany and Nature Protection, University of Warmia and Mazury, 10-728 Olsztyn, Poland; E-Mail: jakub.sawicki@uwm.edu.pl; 2 Department of Biology and Ecology, University of Ostrava, 71000 Ostrava, Czech Republic

**Keywords:** plastid genome, *Pulsatilla*, Ranunculaceae, comparative genomics, barcoding, nuclear rRNA

## Abstract

Background: The European continent is presently colonized by nine species of the genus *Pulsatilla*, five of which are encountered only in mountainous regions of southwest and south-central Europe. The remaining four species inhabit lowlands in the north-central and eastern parts of the continent. Most plants of the genus *Pulsatilla* are rare and endangered, which is why most research efforts focused on their biology, ecology and hybridization. The objective of this study was to develop genomic resources, including complete plastid genomes and nuclear rRNA clusters, for three sympatric *Pulsatilla* species that are most commonly found in Central Europe. The results will supply valuable information about genetic variation, which can be used in the process of designing primers for population studies and conservation genetics research. The complete plastid genomes together with the nuclear rRNA cluster can serve as a useful tool in hybridization studies. Methodology/principal findings: Six complete plastid genomes and nuclear rRNA clusters were sequenced from three species of *Pulsatilla* using the Illumina sequencing technology. Four junctions between single copy regions and inverted repeats and junctions between the identified locally-collinear blocks (LCB) were confirmed by Sanger sequencing. *Pulsatilla* genomes of 120 unique genes had a total length of approximately 161–162 kb, and 21 were duplicated in the inverted repeats (IR) region. Comparative plastid genomes of newly-sequenced *Pulsatilla* and the previously-identified plastomes of *Aconitum* and *Ranunculus* species belonging to the family Ranunculaceae revealed several variations in the structure of the genome, but the gene content remained constant. The nuclear rRNA cluster (18S-ITS1-5.8S-ITS2-26S) of studied *Pulsatilla* species is 5795 bp long. Among five analyzed regions of the rRNA cluster, only Internal Transcribed Spacer 2 (ITS2) enabled the molecular delimitation of closely-related *Pulsatilla patens* and *Pulsatilla* *vernalis*. Conclusions/significance: The determination of complete plastid genome and nuclear rRNA cluster sequences in three species of the genus *Pulsatilla* is an important contribution to our knowledge of the evolution and phylogeography of those endangered taxa. The resulting data can be used to identify regions that are particularly useful for barcoding, phylogenetic and phylogeographic studies. The investigated taxa can be identified at each stage of development based on their species-specific SNPs. The nuclear and plastid genomic resources enable advanced studies on hybridization, including identification of parent species, including their roles in that process. The identified nonsynonymous mutations could play an important role in adaptations to changing environments. The results of the study will also provide valuable information about the evolution of the plastome structure in the family Ranunculaceae.

## 1. Introduction

The family Ranunculaceae consists of 59 genera and approximately 2500 plant species [1], which are distributed worldwide [2] and are commonly found in temperate and cold regions of the Northern Hemisphere. Many taxa of the family Ranunculaceae are pharmaceutically important, and the medicinal value of some species has been confirmed.

One of the genera of the family Ranunculaceae is *Pulsatilla*, which brings together 38 species of herbaceous perennial plants that colonize mainly Europe and Asia. *Pulsatilla* plants have medicinal properties. Recent biological and pharmacological research demonstrated that *Pulsatilla* species contain numerous compounds, including triterpenoid saponins, phytosterols and anthocyanins [3]. The pharmacological effects of these ingredients have not been studied extensively, but protoanemonin was found to exhibit antifungal and antibiotic properties [4,5].

The European continent is presently colonized by nine species of the genus *Pulsatilla* [6], five of which are encountered only in mountainous regions of southwest and south-central Europe. The remaining four species inhabit lowlands in the north-central and eastern parts of the continent. All European species of the genus *Pulsatilla* are rare, endangered and endemic, and their populations are restricted to local habitats. Those taxa are protected on account of their small populations and disappearing localities, and they have been placed on the Red Lists of Endangered Species in all countries they inhabit. In Europe, the genus *Pulsatilla* is represented by light-loving species. Mountainous taxa are found mostly on siliceous bedrock in dry subalpine shrub communities and Alpine grasses, whereas lowland species occupy open habitats in pine forests and dry heathlands. Lowland taxa are often sympatric in their localities, and they can cross-breed and produce hybrids. The most common hybrids are *P. patens* × *P. vernalis* and *P. patens* × *P. pratensis* [7,8,9]. The morphology of pure genotypes has been thoroughly researched, and they are easily distinguished. Hybrids are fertile, but they generally produce fewer seeds; and they can undergo further introgressive hybridization in the maternal population [7].

Most plants of the genus *Pulsatilla* are rare and endangered, which is why most research efforts focused on their biology, ecology and population size [10,11,12,13,14]. The analyzed taxa were also investigated by numerous *in vitro* studies [15,16,17,18,19,20]. Their medicinal properties and applications for alternative medicine were also extensively researched [21,22].

There exists a broad literature dedicated to phylogenetic analyses [23,24,25,26], but most studies focus on phylogenetic relationships between higher taxonomic units (tribe, genera); and this covers the large and varied family Ranunculaceae, which consists of approximately 59 genera and 2500 species [27]. Very few studies discuss phylogenetic relationships between the genus *Pulsatilla* and closely-related genera in the same subfamily Ranunculoideae [28,29]. Genetic resources [30] and the distribution of genetic variation within the geographic range of *Pulsatilla* species have not been widely researched [31]. Most of those taxa are rare and endangered; therefore, their genetic resources should be investigated extensively to contribute to their protection. Information about genetic variation and partitioning of genetic variation across populations and geographical regions of endangered species significantly contributes to the development of conservation strategies and management practices [32]. Due to the haploid nature and low frequency of genetic recombination, molecular markers of organelle DNA have been long used in conservation genetics and analyses of species migratory routes [33,34]. Although chloroplast DNA evolves relatively slowly, moderate to high levels of genetic variation were frequently detected in noncoding spacers within and across species [35,36]. When inherited maternally [37], chloroplast DNA (cpDNA) can be used to investigate processes associated with seed dispersal, such as range expansions [38], and the contribution of seed movement to total gene flow [39]. Chloroplast-specific markers are thus particularly useful for identifying genetic bottlenecks, founder effects and for measuring genetic drift [40]. A thorough knowledge of the rate of those processes in populations of rare and endangered plants contributes to their protection. For this reason, cpDNA sequences are frequently used in conservation genetics [41,42]. Phylogeographic studies were also conducted based on sequences of organelle DNA, including cpDNA [43,44,45,46,47]. Universal primer pairs were most frequently used [48,49]. Presently, genetic variation data for the European species of the genus *Pulsatilla* are available for only two species: *Pulsatilla vernalis* [31] and *Pulsatilla vulgaris* [30].

In many cases, to fully resolve evolutionary and population genetics issues, nuclear DNA regions have to be analyzed [50,51]. The nuclear rRNA cluster demonstrates an unusual pattern of evolution, featuring the interspersion of rapidly-evolving internal transcribed spacers with highly-conserved rRNA genes [52]. Apart from most of the nuclear genes, rRNA genes and spacers are present in thousands of copies located in one or several loci, distributed on one or several chromosomes. Due to hybridization and recombination, divergent copies can exist within the genome [53]. The above features make nuclear rRNA clusters one of the most commonly-used genomic regions in phylogenetic, population genetics, barcoding and hybridization studies [54,55,56,57,58,59].

The objective of this study was to develop genomic resources, including complete plastid genomes and nuclear rRNA clusters of three *Pulsatilla* species (*P. patens*, *P. pratensis* and *P. vernali*s) that are most commonly found in Central Europe. The results will supply valuable information about genetic variation, which can be used in the process of designing primers for population studies and conservation genetics research. Variations in coding regions responsible for the plants’ ability to adapt to stochastic environments are a very important issue in conservation genetics. Information about the chloroplast genome and nuclear rRNA cluster can also facilitate the identification of hybrids, which are common in the genus *Pulsatilla*. The above can be attributed to the fact that chloroplast DNA reveals only half of the parentage in hybrid plants, because it is uniparentaly (primarily maternally) inherited and haploid [60]. To fully resolve hybridization issues, the complete nuclear rRNA cluster (18s rRNA-ITS1-5s rRNA-ITS2-26s rRNA) was assembled. The included internal transcribed spacers (ITS) were successfully used in the previous studies on hybridization in the *Pulsatilla* genus [61].

## 2. Results and Discussion

### 2.1. Size and Structure of Pulsatilla Plastid Genomes

**Figure 1:**
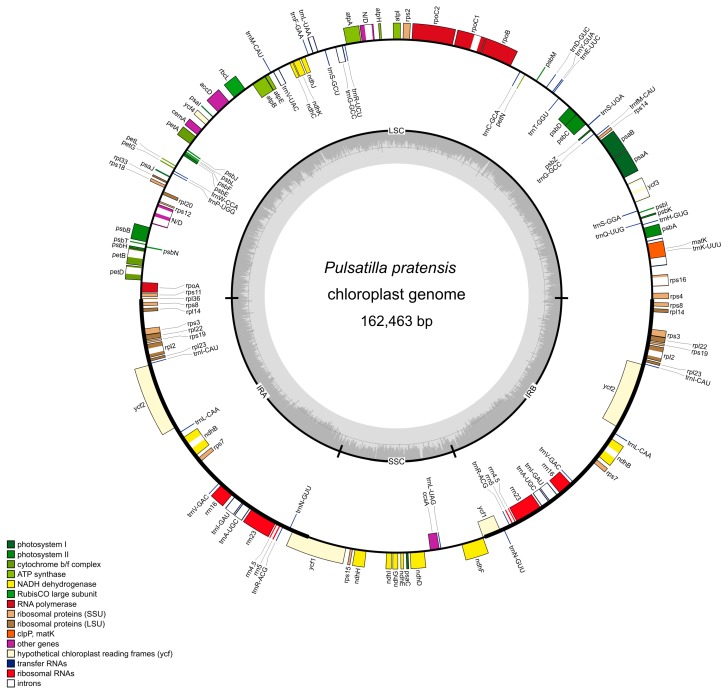
Gene map of the *Pulsatilla pratensis* plastid genome. Genes shown outside the outer circle are transcribed clockwise, and genes inside the outer circle are transcribed counterclockwise. Genes belonging to different functional groups are color coded. The dashed area in the inner circle indicates the guanine-cytosine (GC) content of the plastid genome.

The length of complete plastid genomes of *Pulsatilla* species varied from 161,761 in *P. vernalis* to 162,463 in *P. pratensis* (Figure 1). Minor variations in genome size were also observed at the species level. In *P. patens*, the difference in genome size reached 106 bp, whereas in the remaining species, both genome pairs differed in only 4 bp. The inverted repeats (IR) occurred between *rps*8 and *ycf*1. The genetic content of the analyzed species of the genus *Pulsatilla* was similar to the previously-investigated plastid genomes in the family Ranunculaceae [62].

The *Pulsatilla* plastome encodes 113 genes, excluding the second IR region, which correspond to 79 protein-coding genes, four rRNAs and 30 tRNAs (Table S1). Seventeen of these genes have introns, and one copy of *ycf*1 is a pseudogene. The potential protein-coding genes are *matK*, *ycf3* and *ycf4* in large single copy (LSC), *ycf1* at the small single copy (SSC)-IR border and *ycf2* in IRs.

An analysis of plastid genomes revealed 38, 40 and 47 microsatellite regions in *P. vernalis*, *P. patens* and *P. pratensis*, respectively. In the vast majority of cases, they consisted of 10–16 mononucleotide repeats. Two dinucleotide and nine trinucleotide repeat regions were found in the plastome of *P. pratensis*. In the two remaining species of *Pulsatilla*, only three trinucleotide repeat regions were identified in their plastomes. The total number of identified repeat regions in *Pulsatilla* species was significantly lower than in other analyzed taxa. In the species of the family Ericaceae, the number of simple sequence repeats (SSRs) was determined at 55–62, half of which were mononucleotide repeats [63,64]. In many taxa, the number of microsatellite repeats is 5–6-fold higher [42,65].

An analysis of microsatellite regions in plastid genomes of the investigated *Pulsatilla* species revealed a very similar distribution pattern. In the closely-related *P. patens* and *P. vernalis*, only six and four mononucleotide repeats, respectively, were unique for each species. A higher number of 14 species-specific SSRs was observed in the more genetically-distant *P. pratensis*.

### 2.2. Variation in the Plastid Genome Structure of the Family Ranunculaceae

**Figure 2:**
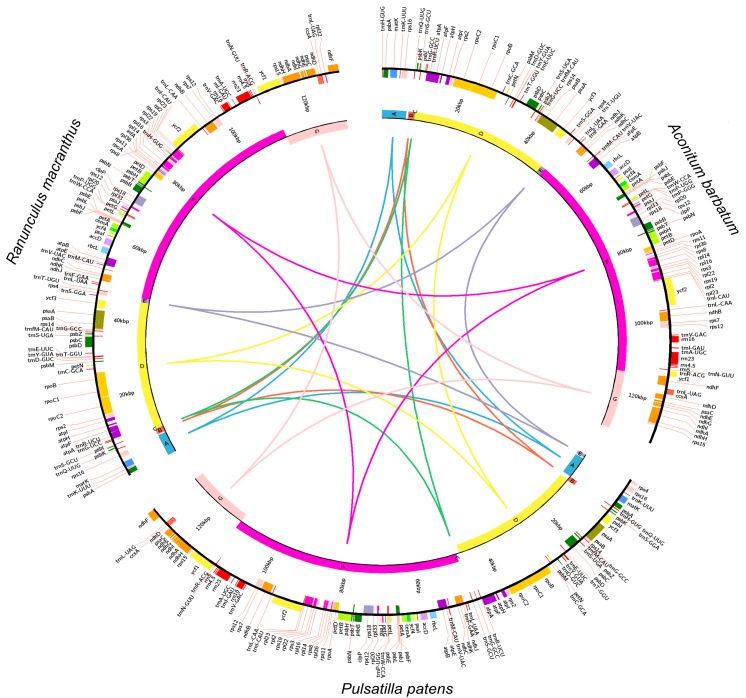
A reciprocal comparison of cpDNA maps of *Aconitum barbatum*, *Ranunculus macranthus* and *Pulsatilla patens*. cpDNA molecules are circular, but they are presented as linear in this figure, excluding the second inverted repeats (IR) region, for easy comparison. Color boxes from the outermost to innermost indicate: (i) genes with counterclockwise transcriptional orientation; (ii) genes with clockwise transcriptional orientation; and (iii) locally-collinear blocks (LCBs), labelled A–G, whose orientation is identical to or opposite of that of *Aconitum*. Orthologous LCBs marked with the same colors and letters are connected by lines. The original, linear Mauve figure is shown in Figure S1.

A comparison of three genomes belonging to different genera of the family Ranunculaceae revealed high variation in plastome structure. Genomes were aligned in the Mauve program to demonstrate seven locally-collinear blocks (LCBs) (Figure 2). The genomic structure changed in both location (Blocks C and E) and orientation (Blocks A and D). The greatest difference relative to the plastid genomes of *Aconitum* and *Ranunculus* is the inversion of the *circa* 37,400-bp region between genes *ycf3* and *trnG-GCC* (Block D) in the LCB.

Structural changes in the plastid genome at the family level are not frequently noted in angiosperms. A comparative genomic analysis revealed a stable plastome structure in 24 species of the family Poaceae [66]. The structure of plastid genomes was nearly identical (small *trnfM* duplication) in the phylogenetically-distant genera *Chamaedaphne* and *Vaccinium* from the family Ericaceae [63,64]. Minor changes involving the inversion of the *petN*-*psbM* region were reported in the family Orchidaceae [42]. By contrast, gymnosperms of the family Pinaceae were characterized by five different plastome structures [67].

The analyzed genomes of *Pulsatilla* species had an identical set of genes to the previously-investigated genomes of *Aconitum* [68] and *Ranunculus* [61]. One of the two copies of *ycf1*, which was produced by incomplete duplication of the normal functional copy of *ycf1* along the IRA/SSC border, was a pseudogene. The second pseudogenized, but much shorter copy of *ycf1* was also located along the IRA/SSC border in the genome of *Ranunculus macranthus*, whereas in the *Aconitum barbatum* genome, both functional copies of *ycf1* were localized in IRs adjacent to SSCs. Similar location and pseudogenization of *ycf1* genes were observed in the genera *Camellia* [69] and *Phoenix* [70].

**Figure 3:**
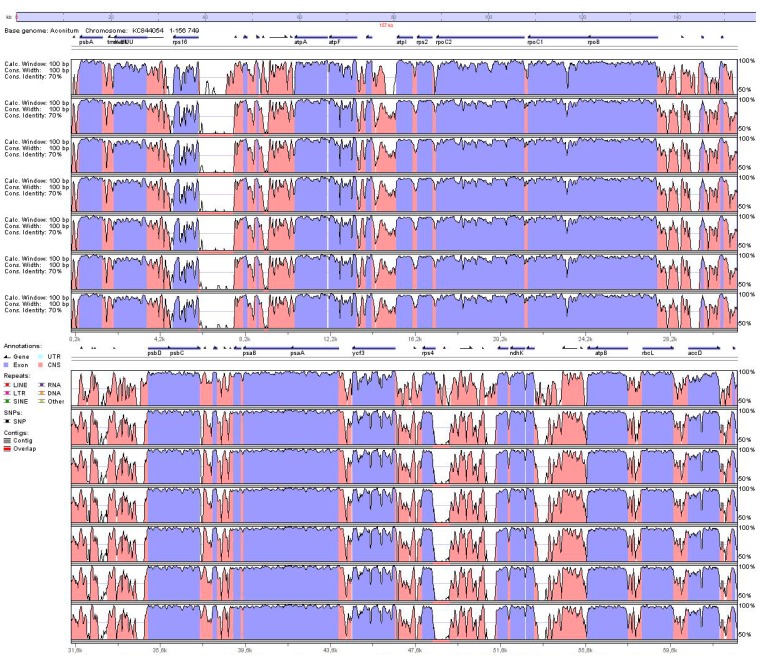

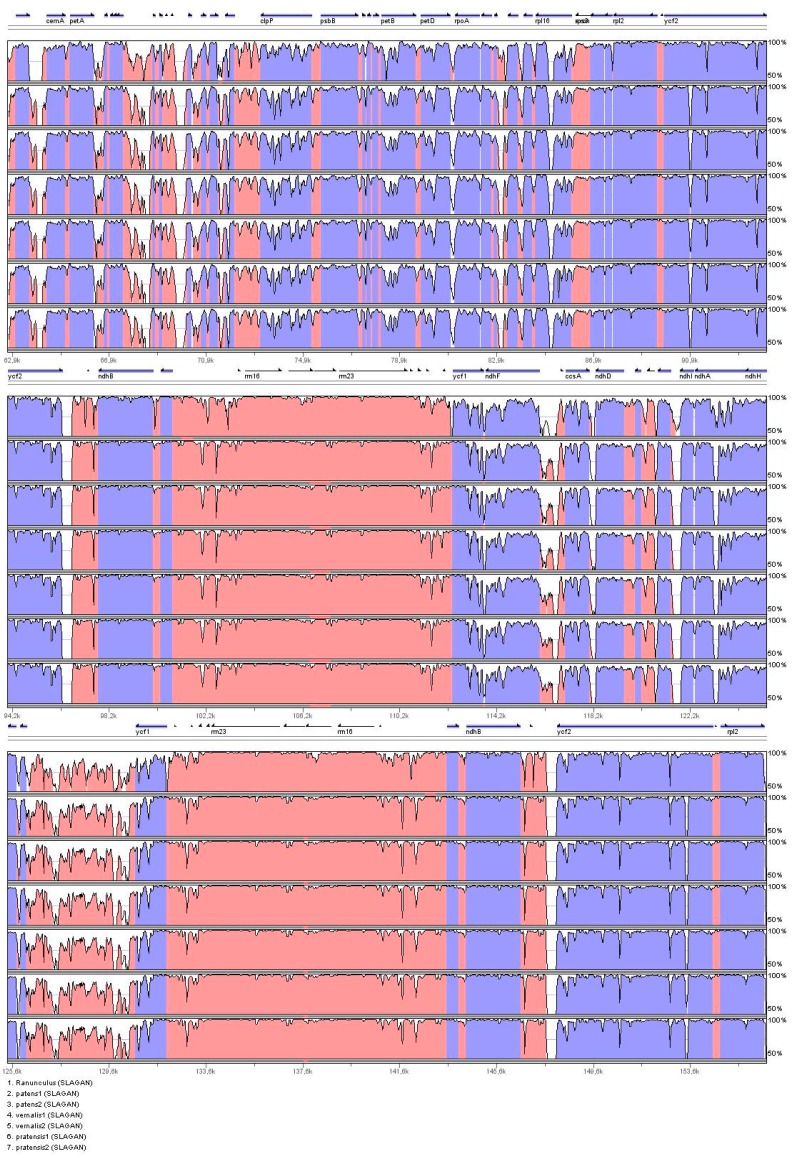
Alignment of eight plastid genome sequences in plants of the family Ranunculaceae. VISTA-based identity plots show sequence identity between six sequenced plastid genomes and the previously-identified sequences in *Ranunculus macranthus* and *Aconitum barbatum* as a reference. The red and purple parts visualize the conserved non-coding and coding regions respectively.

There is moderate genetic divergence in the plastomes sequence between Ranunculaceae species and individuals. Sequence identity was plotted using mVISTA [71] by aligning the seven chloroplast (cp) genomes with *Aconitum barbatum* as a reference (Figure 3). The aligned sequences moderately diverged from more than 30 regions, displaying less than 60% identity, which suggests that cp genomes harbor considerable genetic differentiation, especially in noncoding and single-copy regions. More than 20 divergent hotspot regions were identified.

**Figure 4:**
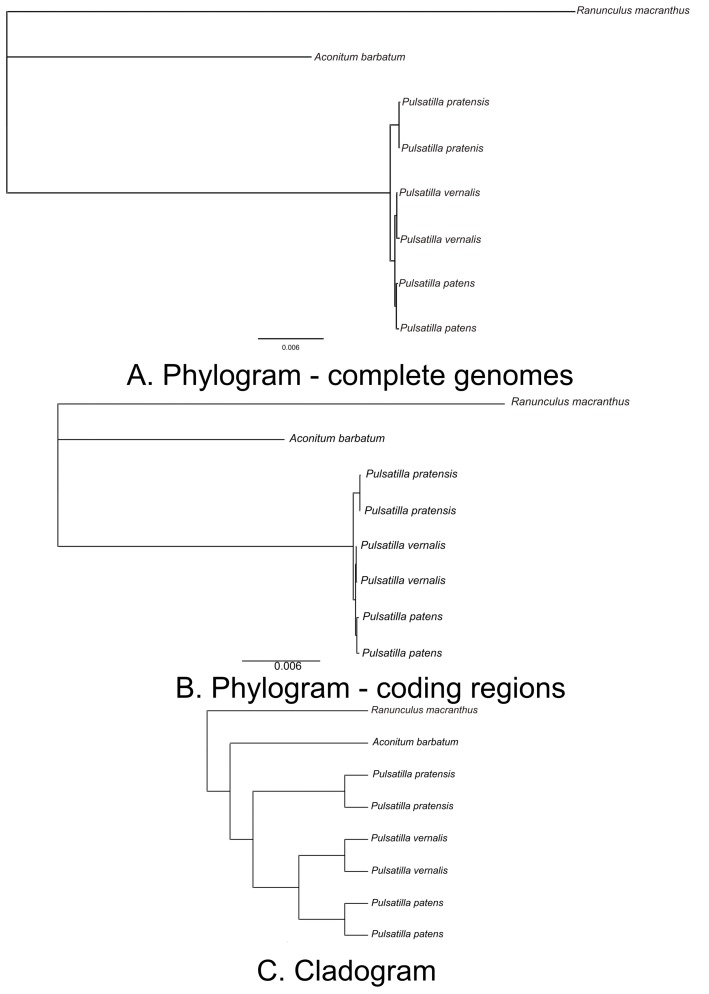
Phylogenetics relationships among studied species based on Bayesian analysis using the general time reversible + gamma (GTR + G) model. All clades are supported by Bayesian interference with posterior probabilities >0.95. (**A**) Phylogram based on complete plastomes; and (**B**) phylogram based on coding regions (CDS), tRNA and rRNA; (**C**) Cladogram given for the better visualization of the relationships among species.

Plastid genome polymorphism patterns revealed close relationships between *P. patens* and *P. vernalis*, which is consistent with previous studies [31]. The phylogenetic trees based on whole plastid genome and coding-only datasets using the Bayesian interference method produced trees with an identical topology, differing only in branch lengths (Figure 4). The analyzed *Pulsatilla* species form a distinct, well-supported clade. *P. vernalis* resolved as a sister to *P. patens*, while *P. pratensis* resolved as a sister to the *patens*/*vernalis* clade.

### 2.3. Identification of the Most Variable Regions of the Pulsatilla Plastid Genome

In lower taxonomic units, there is less variation at the level of the plastid genome, which strongly contributes to differences in hotspot activity. The identification of variations at the genus level is particularly significant in low-level phylogenetics, phylogeography and conservation genetics. In the genus *Pulsatilla*, non-coding regions were characterized by the highest variation in the single-copy regions of the cp genome (Table S2).

Angiosperms clearly tend to accumulate variations at the genus level in regions LSC and SSC of the chloroplast genome. A similar distribution of variations in the plastid genome was reported in the genera *Cymbidium*, *Oenothera* and *Pyrus* [72].

### 2.4. Species-Specific Mutations: Barcoding of Closely-Related Pulsatilla Species

Species-specific mutations (SSM), SNPs and indels were identified in only one species with the consensus sequence as a reference, and no variation was observed at the species level. The highest number of species-specific mutations (SSMs) in plastid genomes was observed in *Pulsatilla pratensis*, whose plastid genome was characterized by 294 substitutions and 138 indels. A much smaller number of SSMs was noted in the remaining two species. The plastid genome of *P. patens* had 49 substitutions and 18 indels, whereas the plastid genome of *P. vernalis* had 41 substitutions and 15 indels, which supported molecular identification. Such significant differences in the number of identified SSMs can be attributed by the phylogenetic position of the analyzed species, where *P. patens* and *P. vernalis* are the most closely-related species [31]. In the group of 125 analyzed regions, 16 had SSMs that enabled the molecular identification of all studied species (Table S2). In many cases, however, the evaluated region would be difficult to examine in routine tests due to its size, which exceeds 1000 bp (e.g., *rps12*-*trnV*, *ndhK*-*trnV*, *ycf1*). Intergenic spacer *ndhD*-*ccsA* deserves special attention due to the number of SSMs and the overall length of the region. The analyzed fragment has a total length of 611 bp, and it contains two indels and five substitutions specific for *P. patens*, nine indels and 18 substitutions specific for *P. pratensis* and two substitutions differentiating *P. vernalis*. Spacer *rps4-rps16* (817 bp), which revealed four substitutions specific for *P. patens*, six substitutions and five indels specific for *P. pratensis*, as well as three substitutions and one indel characteristic of *P. vernalis*, was also highly effective in the molecular identification of species. Another useful region was intron 2 of the *clpP* gene (745 bp), which revealed one indel and two substitutions characteristic of *P. patens*, two indels and five substitutions specific for *P. pratensis* and one indel and one substitution specific for *P. vernalis*. A similar number of SSMs was also noted in the intron of the *rps15* gene (two substitutions and one indel specific for *P. patens*, 10 substitutions and four indels for *P. pratensis* and one indel for *P. vernalis*). The above regions are not part of mainstream barcoding research, but in the few studies where they were analyzed, they showed very high levels of variation [73,74]. In the group of the most commonly-used barcode regions, only *trnH*-*psbA* was characterized by individual mutations (one substitution specific for *P. pratensis* and *P. vernalis*, each, and one indel specific for *P. patens*) that supported molecular discrimination between species.

### 2.5. Mutations in Protein Coding Regions (CDS)

Most variation in the plastid genome is accumulated in spacers and introns that generally exert a neutral effect on the genome [75,76]. In several known cases, the secondary structure of non-coding sequences influenced the expression of the adjacent coding regions and intron mutations near splice sites, which could affect the properties and synthesis of proteins [77,78,79]. Adaptive processes are largely determined by a genome’s coding regions, which should be thoroughly analyzed in endangered species, including the pasque flower. The genomes of six species of the genus *Pulsatilla* were compared to reveal 119 SNP and 10 indel events varying in size from 6 to 39 bp in protein coding regions. In the group of the identified SNPs, 48 were synonymous mutations that do not affect the protein sequence. Nonsynonymous mutations were noted in 71 cases, both within and between species. Nearly half of all nonsynonymous mutations were accumulated in two protein coding genes with unknown function: *ycf1* (29 nonsynonymous mutations) and *ycf2* (five nonsynonymous mutations). Both *ycf1* and *ycf2* are among the most diverse genes in the plastid genome, and they are frequently used in phylogenetics and barcoding [55]. Research into variations in the *ycf1* gene revealed that in a group of 420 tree species, 357 species were discriminated by *ycf1*, which indicates that the analyzed gene has somewhat greater discriminant ability than the combination of *matK* and *rbcL* recommended by Consortium for the Barcode of Life (CBOL) [80].

In the group of genes with known function, five nonsynonymous mutations were observed in the *rpoC2* gene encoding one of the four type-1 RNA subunits. The greatest changes in protein structure were also encoded by the *rpoC2* gene in the genus *Lamium* despite a higher number of mutations in other genes encoding this polymerase [81]. In the remaining genes encoding RNA polymerase in species of the genus *Pulsatilla*, individual nonsynonymous mutations were reported in *rpoA* and *rpoB* genes. Nonsynonymous mutations were also observed in genes encoding small and large ribosome subunits. In this group of genes, the most significant changes were reported in *rpl22*, which was characterized by two nonsynonymous substitutions and an insertion elongating its protein by two amino acids. The remaining genes encoding ribosomal subunits differed in two mutations (*rps14*) or one mutation (*rps15*, *rpl33*), which changed the encoded amino acid. The situation observed in the *ccsA* gene encoding cytochrome C protein was similar to that noted in *rpl22*. The group of genes in cytochrome B/F subunits was relatively conserved, and individual nonsynonymous mutations were observed in *petA*, *petB* and *petD*. In subunits of ATP synthase, nonsynonymous mutations (three) were found only in the *atpA* gene. The genes encoding subunits of NADH dehydrogenase harbored one (*ndhA*, *ndhH*, *ndhK*) or two (*ndhD*, *ndhF*) nonsynonymous substitutions each, and the 39-nucleotide-long insertion in the *ndhF* gene in *P. pratensis* elongated its protein by 13 amino acids. Three and two nonsynonymous mutations were reported in *matK* and *rbcL* genes, respectively, which are frequently recommended for barcoding in plants [82,83]. The *accD* gene, which is popularly used in phylogenetic analyses, had a varied length in plants of the genus *Pulsatilla*, and it was 21 bp longer in the genomes of *P. pratensis*, which elongated the protein sequence by seven amino acids. Only one nonsynonymous mutation was observed in exon 1 of the *clpP* gene encoding a subunit of ATP-dependent protease, despite documented strong positive selection genera *Silene* [84] and *Oenothera* [85].

The analysis of variation in protein-encoding plastid genes indicates that those genes can be effectively used in population and environmental adaptation studies in conservation genetics [86]. The documented positive selection in *rbcL* could possibly be involved in the modulation of RuBisCO aggregation/activation and enzymatic specificity in the family Amaranthaceae [87].

Mutations in several other genes (*accD*, *ccsA*, *matK*, *ndhF*, *rpoC2*) are suspected to play an important role in high altitude adaptation in the genus *Cardamine* [88]. However, in the case of the studied Pulsatilla species, which appear in very similar habitats and climatic conditions, the discovered non-synonymous mutations could play a different role. Despite continuously-grooving resources of available plastome sequences, the adaptations to local habitats are poorly know and published resources.

### 2.6. The Characteristics of the Nuclear rRNA Cluster and Its Usefulness in the Studies on Hybridization

**Figure 5:**
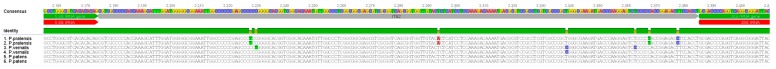
DNA alignment of the ITS2 region of the six studied specimens. The variation and species-specific mutation (SSM) in the nuclear ITS2 region enabled the molecular identification of the three studied species.

The 18s-ITS1-5.8S-ITS2-26s region of the *Pulsatilla* species was 5795 bp long in all studied specimens (the GenBank accession numbers are given in Table S3). Out of 20 detected SNP, 13 were parsimony informative, and seven appeared only in a single sample. The shortest, 164 bp long, 5.8s rRNA gene was monomorphic, and only one SNP specific to *P. pratensis* in the 1810 bp-long 18s rRNA gene was found. Among the six SNPs found in the longest, 3410 bp, 26s rRNA region, five were characteristic for *P. pratensis*, and one was found in the single specimen of *P. vernalis.* The most variable regions of the rRNA nuclear cluster are usually ITS1 and ITS2, commonly used in phylogeny, phylogeography and barcoding studies [89,90]. Despite the relatively short length (200 and 211 bp for ITS1 and ITS2, respectively), these regions accumulated most of the variation of the rRNA cluster. ITS1 was less variable than ITS2, with five detected SNPs, but only two of them could be used for species delimitation (both for *P. pratensis*). Among five regions of the rRNA cluster, only ITS2 enabled a molecular identification of studied *Pulsatilla* species (Figure 5). Similar to the plastid genome, the greatest number of SSMs was found for *P. pratensis* (four), while the molecular delimitation of *P. patens* and *P. vernalis* was made possible on the basis of only one SNP.

The results of rRNA cluster analysis confirmed the advantage of ITS2 over ITS1 in the molecular species identification [75,90]. A similar pattern was observed during studies on Korean *Pulsatilla* species, where only three out of nine total SNPs were detected in the ITS1 region [61]. Our result demonstrated that ITS2 may play an important role in hybridization studies, especially in the case of *P. patens* × *P. vernalis* hybrids.

## 3. Experimental Section

### 3.1. DNA Extraction, Sequencing, Genome Assembly and Annotation

The genomic library of *Pulsatilla patens* was developed based on the results of DNA extraction from a previous study [91]. Total genomic DNA of *P. pratensis* and *P. vernalis* was extracted from fresh leaf tissue using the DNeasy^®^ Plant Mini Kit (Qiagen, Hilden, Germany). Stems were ground with silica beads in a MiniBead-Beater tissue disruptor for 50 s, and they were subsequently processed using the manufacturer’s protocols. DNA quantity was estimated with the use of the Qubit fluorometer system (Invitrogen, Carlsbad, NM, USA) and the Quant-IT ds-DNA BR Assay kit (Invitrogen). From each species, two specimens were sequenced, to check if the discovered SNP can serve as a barcode or represents intraspecific variability.

A genomic library for MiSeq sequencing was developed with the use of the Nextera XT Kit. DNA in the amount of 1 ng was used in the procedure described in the Nextera XT protocol (Illumina, San Diego, CA, USA).

The number and accuracy of libraries was verified with the use of primers whose sequences are given in the Sequencing Library qPCR Quantification Guide (Illumina). PCR reactions were performed in 20 µL of a reaction mixture containing 3 µL of library genomes, 1.0 µM of each primer, 1.5 mM MgCl_2_, 200 µL M dNTP (dATP, dGTP, dCTP, dTTP), 1× PCR buffer and 1 U OpenExTaq polymerase (Open Exome, Warszawa, Poland). PCR reactions were performed under the following thermal conditions: (1) initial denaturation, 5 min at a temperature of 94 °C; (2) denaturation, 30 s at 94 °C; (3) annealing, 30 s at 52 °C; (4) elongation, 1 min at 72 °C and final elongation, 7 min at 72 °C. Stages 2–4 were repeated 34 times. The products of the PCR reaction were separated in the QIAxcel capillary electrophoresis system (Qiagen). Electrophoresis was performed using the QIAxcel High Resolution Kit with the 15–1000-bp alignment marker (Qiagen) and the 25–1000-bp DNA size marker (Qiagen). Standard OL500 settings were used as the electrophoresis program. Validated libraries were pooled according to the Nextera XT protocol. Genomic libraries were sequenced using the MiSeq 500v2 cartridge that supported the acquisition of 2 × 250-bp pair-end reads. The resulting reads were preliminarily assembled using the Velvet *de novo* assembler implemented in the Illumina BaseCloud service.

The obtained contigs were mapped in the *Ranunculus macranthus* (GenBank: NC008796) genome with the use of medium sensitivity settings implemented in the Geneious 6.0.1 software (Biomatters, Auckland, New Zealand). The longest mapped contigs of 6–12 kb were used to reconstruct the *P. patens* genome. All pair-end reads were mapped in the above sequence with the use of a modified medium sensitivity algorithm (with minimum overlap of 100 bp and 99% overlap identity) with 100–1000 iterations. The obtained large contigs were assembled *de novo* using the Geneious 6.0.1 default settings. The flow chart for the *in silico* construction of the *P. patens* plastome was identical to that presented in our previous study [64]. The plastomes of *P. pratensis* and *P. vernalis* were assembled using the same workflow.

The four junctions between single-copy segments and inverted repeats were confirmed using PCR-based product sequencing of the assembled genomes. The sequences of primers used during this step are given in Table S4. Purified PCR products were sequenced in both directions using the ABI BigDye 3.1 Terminator Cycle Kit (Applied Biosystems, Foster City, CA, USA) and visualized with the ABI Prism 3130 Automated DNA Sequencer (Applied Biosystems). The sequences obtained with the Sanger method were aligned with the assembled genomes using the Geneious 6.0.1 assembly software to check for any differences.

The assembled plastid genomes were annotated with DOGMA [92] and Geneious 6.0.1. The coordinates of exons and introns were manually checked by aligning the respective genes of *Ranunculus macranthus* (GenBank: NC008796) and *Aconitum barbatum* (GenBank: KC844054). The specimens and sequencing details with GenBank numbers are given in Table S3.

The genomes of *Aconitum*, *Pulsatilla patens* and *Ranunculus* were lined up in the Mauvee program [93] to determine changes in plastome structure. Changes in the orientation and placement of identified blocks were visualized in the Circos application [94].

The sequences of plastomes without a second IR region were scanned for microsatellite motifs with the use of MSTATCOMMANDER [95]. The minimal number of mononucleotide repeats was set to 10, dinucleotide repeats to six and trinucleotide repeats to four.

*De novo* assembly of the nuclear rRNA region was with the obtained mapped contigs in the ITS sequence of *Pulsatilla patens* (GenBank: AM267280) with the use of medium sensitivity settings implemented in the Geneious 6.0.1 software. All pair-end reads were mapped in the above sequence with the use of a modified medium sensitivity algorithm (with a minimum overlap of 100 bp and 99% overlap identity) with 200 iterations. The assembled nuclear rRNA clusters were annotated based on BLAST results using Geneious 6.0.1. The annotated rRNA clusters were aligned using the Multiple Sequence Alignment Tool (MUSCLE) [96].

### 3.2. Selection of Plastid Regions as Markers for Evolutionary Studies of Pulsatilla

All regions, including coding regions, introns and intergenic spacers, were extracted to identify divergence regions within six *Pulsatilla* plastid genomes for phylogenetic applications. Every homologous region was aligned using the Multiple Sequence Alignment Tool (MUSCLE) [96], and additional manual adjustments were performed where necessary. The percentage of variable characters within each region was calculated.

The number of nucleotide substitutions and indels (potentially informative characters (PIC)) between the six plastid genomes was tallied for each cpDNA region. Indels were scored in this study because they have been shown to be prevalent and often phylogenetically informative [89,97,98]. Indels and any nucleotide substitutions within indels were scored as independent, single characters. Three types of calculations were performed. Firstly, the proportion of mutational events for each noncoding cpDNA region was estimated based on the formula proposed by O’Donnell [99].

### 3.3. Resolving Phylogenetic Relationships

The studied plastid genomes were aligned using the Mauvee genome aligner [93], excluding the second IR repeat. For completed plastid genomes, as well as coding-only alignment, the parameters of the likelihood model were those of the general time reversible model with gamma distributed rate variation among sites (General time reversible model (GTR) + Gamma model (G)) in accordance with the best fitted nucleotide evolution model selected on the basis of the Akaike information criterion [100] scores in Modeltest 3.7 [101].

Bayesian analysis was carried out in MrBayes 3.2 [102]. The MCM algorithm was run for 4,000,000 generations with four incrementally-heated chains, starting from random trees and sampling one out every 1000 generations. Tracer 1.3 [103] was used to examine the parameters and to determine the number of trees needed to reach stationarity (burn-in), which occurs at around 40,000 generations, and therefore, the first 50 trees were discarded as burn-in. The remaining trees were used to develop a Bayesian consensus tree.

## 4. Conclusions

An analysis of variation in the genomes of three closely-related, sympatric species of the genus *Pulsatilla* revealed cpDNA regions that can be effectively used in phylogenetic, phylogeographic and population genetic studies. The investigated species can be successfully identified based on several regions of the plastid genome, as well as based on the nuclear ITS2 region. Our findings can prove to be highly useful in the research of the hybridization in the genus *Pulsatilla*. A comparative analysis of plastomes in the species of the family Ranunculaceae revealed a high rate of changes in genomic structure, but only minor differences in gene content.

## Supplementary Materials

Supplementary materials can be found at http://www.mdpi.com/1422-0067/16/09/22258/s1.
