# Supplementary material for: Genomic Resources of Three Pulsatilla Species Reveal Evolutionary Hotspots, Species-Specific Sites and Variable Plastid Structure in the Family Ranunculaceae
Source: Int J Mol Sci. 2015 Sep 15;16(9):22258–79. doi: 10.3390/ijms160922258 (PMC4613307; doi:10.3390/ijms160922258)
Supplement: Supplementary file 1 [file ijms-16-22258-s001.zip › ijms-89872-Supplementary Information/ijms-89872-Table S1 and Figure S1.pdf]

Supplementary Information

Table S1. Genes found in the plastid genome of *Pulsatilla*.

Type of Gene	Gene Names
Acetyl-CoA carboxylase	*accD* [a]
ATP synthase subunits	*atpA* [a], *atpB* [a], *atpE* [a], *atpF* [a], *atpH* [a], *atpI* [a]
Cytochrome B/F complex subunit	*petA* [a], *petB* [a], *petD* [a], *petG* [a], *petL* [a], *petN* [a]
Cytochrome C protein	*ccsA* [a]
Envelope membrane protein	*cemA* [a]
Maturase K	*matK* [a]
NADH-dehydrogenase subunits	*ndhA* [a], *ndhB* [c], *ndhC* [a], *ndhD* [a], *ndhE* [a], *ndhF* [a], *ndhG* [a], *ndhH* [a], *ndhI* [a], *ndhJ* [a], *ndhK* [a]
Photosystem I subunits	*psaA* [a], *psaB* [a], *psaC* [a], *psaI* [a], *psaJ* [a]
Photosystem II subunits	*psbA* [a,d], *psbB* [a], *psbC* [a], *psbD* [a], *psbE* [a], *psbF* [a], *psbH* [a], *psbI* [a], *psbJ* [a], *psbK* [a], *psbL* [a], *psbM* [a], *psbN* [a], *psbT* [a], *psbZ* [a]
Ribosomal RNAs	*rrn16* [c], *rrn23* [c], *rrn4.5* [c], *rrn5* [c]
RNA polymerase subunits	*rpoA* [a], *rpoB* [a], *rpoC1* [a], *rpoC2* [a]
Rubisco large subunits	*rbcL* [a]
Small ribosomal protein units	*rps2* [a], *rps3* [c], *rps4* [a], *rps7* [c], *rps8* [c], *rps11* [a], *rps14* [a], *rps15* [a], *rps16* [a], *rps18* [a], *rps19* [c]
Large ribosomal protein units	*rpl2* [c], *rpl14* [c], *rpl16* [c], *rpl20* [a], *rpl22* [c], *rpl23* [c], *rpl33* [a], *rpl36* [a]
Hypothtical genes	*ycf1* [cd], *ycf2* [c], *ycf3* [a], *ycf4* [a]
Transfer RNAs	*trnA-UGC* [c], *trnC-GCA* [a], *trnD-GUC* [a], *trnE-UUC* [a], *trnF-GAA* [a], *trnG-GCC* [a], *trnG-UUC* [a], *trnH-GUG* [c], *trnI-CAU* [c], *trnI-GAU* [c], *trnK-UUU* [a], *trnL-CAA* [c], *trnL-UAA* [a], *trnL-UAG* [a], *trnMf-CAU* [a], *trnN-GUU* [c], *trnP-GGG* [a], *trnP-UGG* [a], *trnQ-UUG* [a], *trnR-ACG* [c], *trnR-UCU* [a], *trnS-GCU* [a], *trnS-GGA* [a], *trnS-UGA* [a], *trnT-GGU* [a], *trnV-GAC* [c], *trnV-UAC* [a], *trnW-CCA* [a], *trnY-GUA* [a]
Other genes (translational initiation factor A and photosystem II protein Z, respectively)	*clpP* [a], *infA* [a,b], *lhbA* [a]

[a] Genes located in single-copy regions (SC); [b] Pseudogene; [c] Gene located in the inverted repeats (IR); [d] Two copies, one functional and one pseudogene.

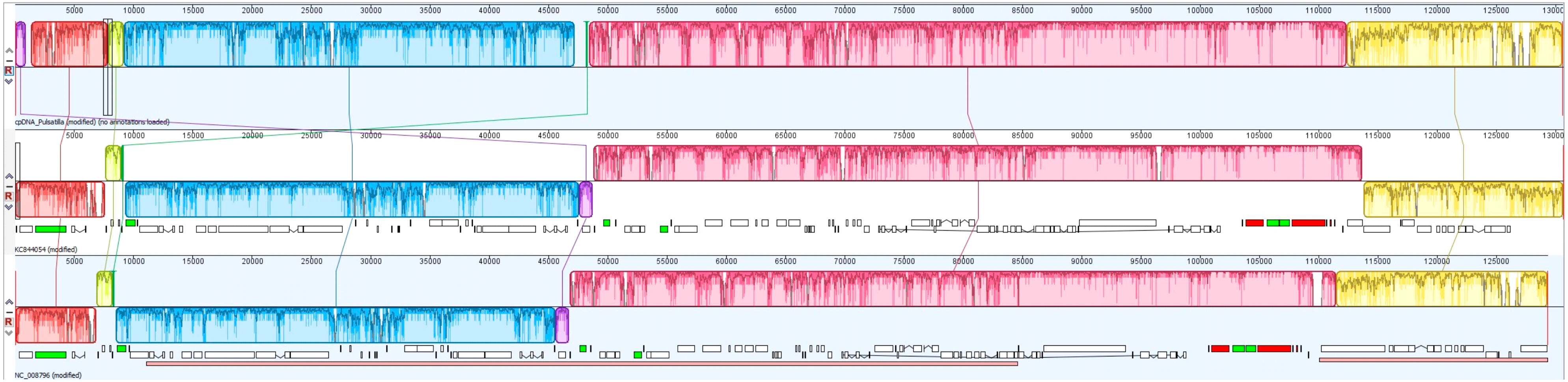

Figure S1. Mauvee alingmnet of the three Ranunculaceae genomes. Each of locally collinear blocks is coded in different color.
